# Myocarditis associated with campylobacter jejuni colitis: a case report

**DOI:** 10.11604/pamj.2020.36.199.23370

**Published:** 2020-07-21

**Authors:** Oussama Daboussi, Salamata Diallo, Boundia Djiba, Mamadou Ngoné Gueye

**Affiliations:** 1 Department of Gastroenterology, Hospital Center Chartres, France,; 2 Department of Gastroenterology, Hospital Center Aristide Le Dantec, Senegal,; 3 Department of internal Medicine, Hospital Center Aristide Le Dantec, Senegal,; 4 Department of Gastroenterology, Hospital Center Idrissa Pouye, Senegal

**Keywords:** Campylobacter jejuni, colitis, myocarditis

## Abstract

Myocarditis is a rare complication of acute diarrhea due to Campylobacter Jejuni infection. We present the case of 25-year-old male who presented with campylobacter jejuni colitis who subsequently had chest pain and elevated cardiac biomarkers. The patient developed acute myocarditis confirmed on cardiac magnetic resonance imaging.

## Introduction

Myocarditis is a rare condition that can mimic an acute coronary syndrome. It can develop as a complication of different viral infections. Myocarditis is seldom associated with bacterial source. We report a case of myocarditis confirmed on cardiac MRI related to campylobacter jejuni colitis.

## Patient and observation

**Figure 1:**
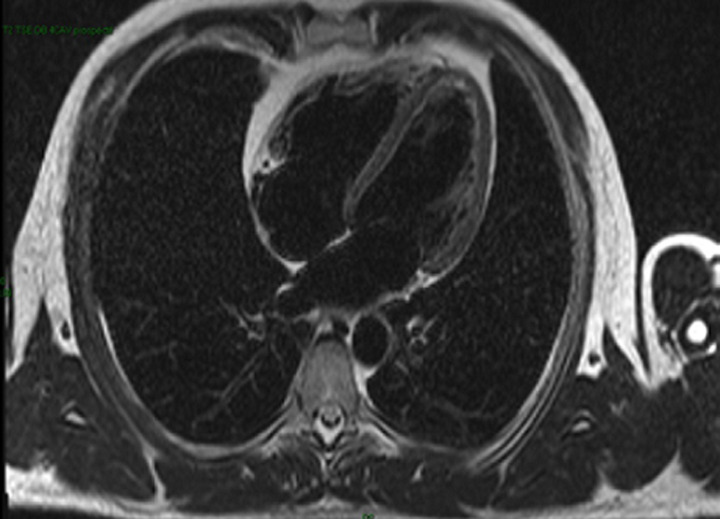
four-chamber view of the left ventricle: increased signal intensity of the lateral wall on T2 weighted images indicating the presence of myocardial edema

**Figure 2:**
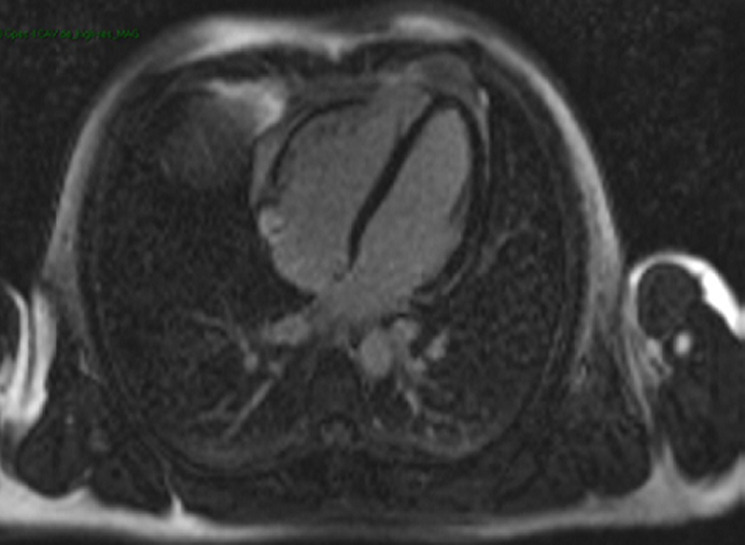
phase Sensitive Inversion Recovery (PSIR) on T1 weighted images: subepicardial minor delayed enhancement after administration of gadolinium

A 25-year-old previously healthy man with no cardiac risk factor was admitted with three days history of acute watery diarrhea, nausea and fever associated with abdominal pain. A few days before the onset of diarrhea, he had taken non-steroidal anti-inflammatory drugs for lower back pain. Campylobacter jejuni resistant to ofloxacin was recovered from the stool culture. He was commenced on erythromicin 2g daily. Two days after admission, he developed chest pain with no radiation. He had no associated breath shortness or palpitations. This was his second such episode in two days. On admission he was chest pain-free and the electrocardiogram (ECG) was normal. Laboratory investigations revealed normal blood counts and serum electrolytes. C-reactive protein was 85 mg/L (reference < 5). Total creatine kinase (CK) was elevated to 684 U/L (reference range 20-200), troponin I was 136 ng/mL (reference range 0-14). ECG showed a sinus rhythm without ST segment changes. Echocardiography showed normal systolic function with a left ventricular ejection fraction (LVEF) of 57% with no evidence of pericarditis. Cardiac magnetic imaging confirmed reduced LV systolic function (45%) and showed areas of increased signal intensity on T2-weighted images suggesting myocardial edema (Figure 1). After administration of intravenous gadolinium inconspicuous delayed enhancement of the lateral wall was seen (Figure 2). Low B-blockers were initiated with angiotensin converting-enzyme inhibitors. The diagnosis was acute myocarditis associated with campylobacter jejuni infection. On day four, the troponin I and CPK decreased to 93 U/L and 87ng/mL respectively. The patient was discharged from the hospital in a stable condition.

## Discussion

This is a rare case of myocarditis associated with Campylobacter enteritis in young male patient. Myocarditis is an inflammatory heart muscle disease associated with cardiac dysfunction. In most cases, enterovirus and adenovirus infections are the frequent cause of myocarditis. Campylobacter-associated myocarditis is a very rare and potentially life-threatening complication of C. Jejuni enterocolitis with very small number of cases reported [1-3]. The diagnosis of myocarditis is established by a combination of clinical, laboratory and cardiac imaging criteria. The diagnosis should be contemplated when a patient presents with unexplained congestive heart failure chest pain and elevated cardiac enzyme levels in the absence of coronary disease, one- or two-weeks following symptoms of gastroenteritis. ECG findings may include arrhythmias, atrioventricular block and nonspecific repolarization abnormalities. ECG can also be normal [4]. Cardiac magnetic resonance imaging is very useful as a non-invasive method in cases of suspected myocarditis. It looks for evidence of new or recent myocardial damage, increased T2 signal or delayed enhancement [5,6]. The physiopathology of myocarditis in human is not well understood, several theories have been developed. The short time interval between the onset of colitis and the onset of myocarditis make a post-infection autoimmune mechanism, which can result in myocyte damage, unlikely. C jejuni associated cardiac involvement may be mediated by a direct bacterial insult to cardiac tissue, toxin, or cytotoxic T-cells. Although C. Jejuni produces several exotoxins with hemolytic, hepatotoxic, and cytotoxic effects, none are known to be toxic to the heart [7,8].

## Conclusion

Despite being a rare complication, C. Jejuni associated myocarditis should be suspected when chest pain with elevated cardiac enzymes occur shortly after an episode of diarrhea and fever. Given the prevalence of Campylobacter Jejuni colitis we recommend that physicians be aware of the potential sequelae of this bacterium.
